# Multicentre randomised controlled trial to evaluate the efficacy of DIALIVE 2.0, a liver dialysis device, for the treatment of acute-on-chronic liver failure: study protocol

**DOI:** 10.1136/bmjopen-2026-125155

**Published:** 2026-11-06

**Authors:** Sina Jameie-Oskooei, Rohit Saha, Sameer Patel, Mansoor N Bangash, Gautam Mehta, Carrie Morgan, Juan-Antonio Carbonell, Banwari Agarwal, Rajiv Jalan

**Affiliations:** 1 Institute for Liver and Digestive Health, University College London, London, UK; 2 Critical Care Unit, Royal Free Hospital, London, UK; 3 Critical Care Unit, King’s College Hospital, London, UK; 4 Liver Intensive Therapy Unit, King’s College Hospital, London, UK; 5 Critical Care Unit, Queen Elizabeth Hospital, Birmingham, UK; 6 Department of Inflammation and Ageing, University of Birmingham, Birmingham, UK; 7 Birmingham Liver Failure Research Group, Birmingham, UK; 8 Yaqrit Ltd, London, UK; 9 INCLIVA Health Research Institute, Valencia, Spain

**Keywords:** Hepatology, Adult intensive & critical care, Randomized Controlled Trial

## Abstract

**Introduction:**

Acute-on-chronic liver failure (ACLF) is a syndrome characterised by acute decompensation of cirrhosis, systemic inflammation and multiorgan failure, and is associated with high short-term mortality. Current management is largely supportive, with emergency liver transplantation available to only a small minority of patients. DIALIVE is a novel extracorporeal liver support device designed to modulate systemic inflammation and restore albumin function. The first-in-human multicentre European trial established a reasonable safety profile and showed evidence of the intended biological activity. The present study aims to evaluate the efficacy and safety of an updated system, DIALIVE 2.0, in a larger, multicentre randomised controlled trial.

**Methods and analysis:**

This multicentre, open-label, randomised controlled trial will enrol 72 adults with persistent A-TANGO ACLF grades 2–4 despite at least 48 hours of standard of care (SoC) across approximately 12 UK centres. Participants will be randomised 1:1 to SoC alone or SoC plus up to seven sessions of DIALIVE 2.0 over a 10-day treatment window. The primary endpoint is the proportion of participants achieving ACLF resolution by day 10. Secondary endpoints include 28-day and 90-day transplant-free survival, time to intensive care unit and hospital discharge, resolution of individual organ failures and safety outcomes including serious adverse events. Efficacy analyses will be conducted on an intention-to-treat basis, with a prespecified fixed-sequence testing procedure for the primary endpoint and the key secondary endpoint of 28-day transplant-free survival.

**Ethics and dissemination:**

Favourable opinion was obtained from the London–South East Research Ethics Committee (26/LO/0090), the Health Research Authority has granted approval and the Medicines and Healthcare products Regulatory Agency (MHRA) has issued a ‘no objection’ decision (CI/2025/0075/GB). An independent Data Safety Monitoring Board will oversee participant safety and trial conduct. Informed consent will be obtained from participants, or from a personal/legal representative under the Mental Capacity Act 2005 (or equivalent legislation) where the participant lacks capacity. Results will be disseminated through peer-reviewed publications, conference presentations and appropriate data-sharing mechanisms.

**Trial registration number:**

NCT07347275.

**STRENGTHS AND LIMITATIONS OF THIS STUDY**

- Concealed electronic allocation reduces selection bias, while the multicentre design supports generalisability across participating clinical settings.
- Prespecified intention-to-treat analyses will be conducted by an independent statistician blinded to treatment allocation.
- Serial assessment of biomarkers of systemic inflammation, albumin function, cell death and kidney injury enables evaluation of biological changes alongside clinical outcomes.
- Blinding is not feasible because the control group does not undergo sham extracorporeal therapy, introducing potential performance and ascertainment bias.
- The planned sample size of 72 participants and restrictive eligibility criteria may limit the certainty of the estimated treatment effect and the generalisability of findings to the broader acute-on-chronic liver failure population.

## Introduction

 Acute-on-chronic liver failure (ACLF) is a distinct and severe condition that develops in patients with underlying cirrhosis. It is defined by acute hepatic decompensation that typically follows a pro-inflammatory precipitating event, leading to intense systemic inflammation and multiorgan dysfunction.^1^ ACLF has been observed at presentation in over 20% of patients hospitalised for acute decompensation of cirrhosis, with a further 8% developing ACLF during admission.^2^ Large international cohort studies have consistently confirmed that ACLF carries a high short-term mortality.^2–4^

Intensive care, organ support and prompt treatment of precipitating events remain the mainstay of ACLF management. Liver transplantation is an emerging option for selected patients with advanced ACLF but remains a scarce resource and is precluded in many patients by comorbidities, advanced age or severity of acute illness. Consequently, there has been growing interest in extracorporeal liver support (ECLS) therapies, predicated on their immunomodulatory and regenerative potential. The most extensively studied artificial ECLS systems, MARS (Molecular Adsorbent Recirculating System), which uses albumin dialysis and Prometheus, which uses fractionated plasma separation and adsorption, as well as the bioartificial ELAD (Eextracorporeal Lliver Aassist Ddevice) have not demonstrated a survival benefit in ACLF in randomised controlled trials.^5–7^ The absence of an effect on overall and transplant-free survival with MARS and Prometheus has been confirmed in a network meta-analysis.^8^

DIALIVE is an ECLS device designed to address several of the key pathophysiological drivers of ACLF, including systemic inflammation, immune dysfunction and loss of albumin function. It works by removing pathogen-associated molecular patterns (PAMPs), damage-associated molecular patterns (DAMPs) and dysfunctional albumin. DIALIVE has the potential to improve outcomes by promoting ACLF resolution and improving clinically relevant endpoints including transplant-free survival. Phase 1 and 2 studies^9 10^ have demonstrated its safety and biological activity, including improvement in albumin function, reduction in systemic inflammation and accelerated ACLF resolution.

The safety and biological activity of DIALIVE were evaluated in a randomised, controlled trial in patients with ACLF precipitated by alcohol-related hepatitis.^10^ 32 patients received up to 5 days of DIALIVE treatment, with outcomes assessed at day 10. DIALIVE was found to be safe, with no significant difference in overall serious adverse event (SAE) counts compared with controls, although events related to hypotension and coagulopathy were more frequent in the treatment arm. Among patients receiving at least three DIALIVE sessions, significant improvements were observed in albumin function, systemic inflammation and biomarkers of cell death and endothelial dysfunction. DIALIVE was associated with faster resolution of ACLF and improvement in prognostic scores. The study was not designed or powered to evaluate 28-day mortality. These findings supported the safety profile of DIALIVE and suggested potential clinical benefit, warranting further investigation in an adequately sized clinical trial.

## Rationale for DIALIVE 2.0

DIALIVE 2.0 incorporates a commercially available plasmapheresis platform (Medica AFERsmart Plus system) that regulates flow rates, treatment duration and pressures, together with a dual-filtration cartridge arranged in series (figure 1). The first filter (Alteco LPS Adsorber) adsorbs PAMPs such as endotoxin and DAMPs. The second filter (Medica FRACTIOsmart Small 1.0) is a high-cut-off membrane that allows ultrafiltration of albumin and cytokines. Filtered albumin is replaced with a predetermined volume of 20% human albumin solution to maintain oncotic stability and restore albumin function. The modified filters and shorter treatment duration incorporated into DIALIVE 2.0 are anticipated to reduce hypotensive and coagulopathy related events and to improve efficacy further. This combined treatment concept—Selective Plasma filtration, Endotoxin Adsorption and Albumin Replacement (SPEAR)—encapsulates the three core mechanisms underpinning DIALIVE 2.0’s mode of action.

**Figure 1:**
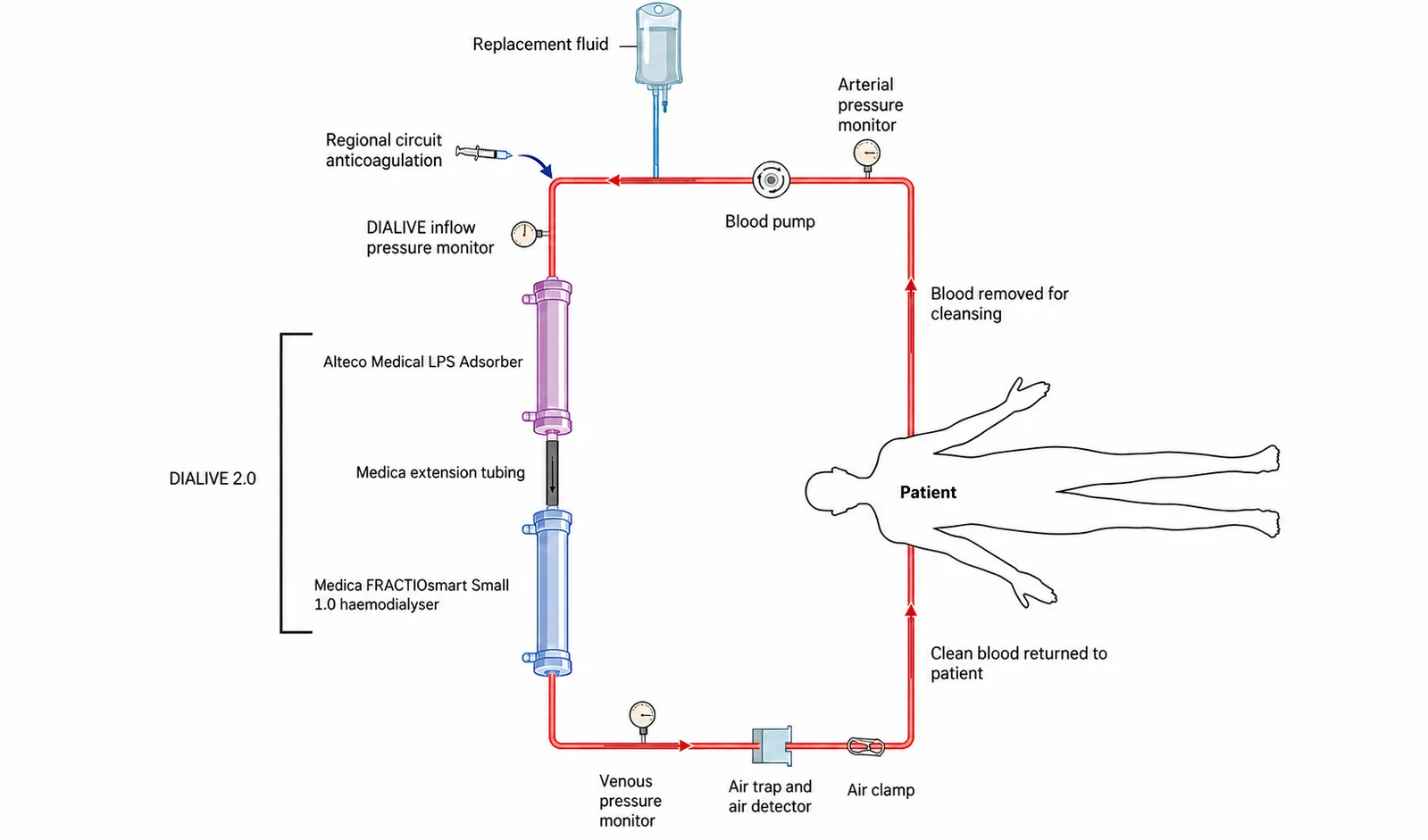
Schematic of the DIALIVE 2.0 extracorporeal liver-support circuit.

## Methods

### Study design

This is a multicentre, randomised, controlled, open-label, parallel-group trial with two arms designed to evaluate the efficacy and safety of DIALIVE 2.0 when added to standard management of patients with A-TANGO ACLF grades 2–4. The control arm receives standard of care (SoC) only. The experimental arm receives SoC plus up to seven daily DIALIVE 2.0 sessions during a 10-day treatment window. The trial is designed to test the superiority of DIALIVE 2.0 plus SoC over SoC alone for the primary efficacy endpoint.

### Participant selection

The trial will recruit 72 inpatients from approximately 12 hospitals across the UK. Eligible participants are adults aged 18–70 years with a diagnosis of A-TANGO ACLF grades 2–4, a history of liver cirrhosis and an acute deterioration attributable to a precipitating event culminating in ACLF. The full inclusion and exclusion criteria are outlined in table 1.

**Table 1:** Eligibility criteria

Eligibility category	Criterion
Inclusion	Adults aged 18–70 years
	Diagnosis of A-TANGO ACLF grades 2–4 (according to A-TANGO ACLF criteria)
	Persistent A-TANGO ACLF grades 2–4 despite receipt of SoC for at least 48 hours
	Inclusion within 10 days of the onset of A-TANGO ACLF grade ≥2
Exclusion	A-TANGO ACLF ≥grade 2 for more than 10 days prior to inclusion
	Pregnancy
	Co-infection with HIV/AIDS, defined by a CD4+T cell count <200 cells/µL, a CD4+T cell percentage of total lymphocytes <15%, or an AIDS-defining illness (eg, Pneumocystis jirovecii pneumonia, Kaposi’s sarcoma, cytomegalovirus disease, invasive candidiasis)
	Bacterial infection or sepsis unresponsive to 48 hours of antimicrobial therapy and/or worsening haemodynamic instability
	Invasive fungal infection (clinical or radiological evidence; not solely biomarker positivity such as beta-D-glucan or galactomannan)
	Acute or subacute liver failure in the absence of cirrhosis
	Posthepatectomy liver failure or primary non-function following liver transplantation
	Previous liver transplant
	Severe thrombocytopaenia (platelet count <20×10⁹/L at screening) or evidence of rapid decline in platelet count (>50% reduction within the preceding 24 hours)
	International normalised ratio >3.0, unless sustained correction for >24 hours following fresh frozen plasma or prothrombin complex concentrate
	Severe disseminated intravascular coagulation
	Persistent haemodynamic instability, defined as: (1) norepinephrine dose >0.5 µg/kg/min; (2) norepinephrine-equivalence index >0.5 µg/kg/min if a second vasopressor is used or (3) arterial lactate >4 mmol/L despite 24 hours of adequate fluid resuscitation and vasopressor therapy
	Severe respiratory failure: PaO₂/FiO₂ ≤150 mm Hg (20 kPa)
	Established on renal replacement therapy for >48 hours prior to inclusion
	A-TANGO ACLF-WBC score >64 (formula provided in online supplemental file 2)
	Significant and/or uncontrolled bleeding (participants may be enrolled 48 hours after bleeding is controlled)
	Active or prior history of non-hepatic malignancy unless adequately treated or in complete remission for 5 or more years
	Hepatocellular carcinoma outside of Milan criteria
	Acute, extensive, portal vein thrombosis extending to and occluding the superior mesenteric vein
	Major systemic illness, aside from liver disease, that in the opinion of the investigator would preclude the patient from participating in the study
	Pre-existing chronic kidney disease defined as estimated glomerular filtration rate <30 mL/min for 3 months or longer prior to screening
	Severe frailty (Clinical Frailty Scale >5)
	Severe malnourishment (BMI <18 kg/m^2^)
	Participants not considered appropriate for full active treatment including organ support
	Participants who have a ‘do not attempt cardio-pulmonary resuscitation’ order in place
	Participants who have received an investigational drug or device within 30 days, or who are scheduled to receive another investigational drug or device during the study
	Uncontrolled seizures
	Evidence of new intracranial pathology such as cerebral haemorrhage or infarction
	Participants diagnosed with Creutzfeldt-Jakob disease
	Participants unable to consent for themselves, unless a legal representative/consultee is appointed and approved as dictated by local and national legislation
	Known allergy to heparin, or type II heparin-induced thrombocytopenia
	In the opinion of the investigator, recruitment to the study would be unsafe for the participant

ACLF, acute-on-chronic liver failure; BMI, body mass index; FiO₂, fractional inspired oxygen; PaO₂, arterial oxygen partial pressure; SoC, standard of care; WBC, white blood cell count.

### Participant recruitment

The study will commence in August 2026 and is currently scheduled for completion in August 2027. However, an extension may be sought depending on recruitment progress and subject to the necessary approvals. Patients must be enrolled within 10 days of the onset of A-TANGO ACLF grade ≥2. All patients are expected to receive at least 48 hours of optimal SoC before randomisation, to confirm that the ACLF is not resolving with standard treatment. This requirement applies regardless of presenting grade, on the basis that ACLF that progresses or plateaus despite SoC defines the population for whom an adjunctive ECLS therapy is most likely to be of benefit. In the first-in-human DIALIVE trial the precipitating event was alcohol-related hepatitis in all participants. However, DIALIVE 2.0 will not restrict recruitment by precipitant aetiology.

Non-response to SoC will be assessed by repeat A-TANGO organ-failure assessment after at least 48 hours of SoC. Participants will be considered non-responsive if they continue to meet criteria for A-TANGO ACLF grade 2–4 at reassessment. The recently described A-TANGO Organ Failure (A-TANGO OF) score^11^ (online supplemental file 1) was selected to guide recruitment because it may more accurately identify and stratify patients with ACLF than existing criteria. By refining organ-dysfunction thresholds and incorporating ACLF grade 4, it broadens the clinically relevant population and increases trial eligibility.

### Sample size

Sample-size estimates were informed by data from the first-in-human DIALIVE trial,^10^ which suggested an expected day-10 ACLF resolution rate of 20% in the SoC arm and 55% in the DIALIVE 2.0 arm. Accepting a two-sided α of 0.05, β of 0.20 (ie, 80% power) and an anticipated total attrition of 10% (5% drop-out and 5% censoring for liver transplantation within 28 days), a total of 72 participants (36 per arm) is required. Sample size was calculated using the arcsine approximation.

### Randomisation

Eligible participants will be individually randomised 1:1 to SoC or SoC plus DIALIVE 2.0 via the electronic case report form (eCRF) based computerised system. A site-specific random allocation sequence will be generated automatically. The allocation sequence will be concealed within the eCRF system and will not be accessible to recruiting investigators before assignment. Given the nature of the intervention, the trial is open-label and neither participants nor treating clinical teams will be blinded to treatment allocation, as the use of sham extracorporeal therapy in the control arm was considered ethically unacceptable. Treatment allocation will only be revealed after eligibility has been confirmed and the participant has been entered into the randomisation system. To minimise analytical bias, prespecified statistical analyses will be conducted by an independent statistician blinded to treatment allocation.

### Consent

Written informed consent will be obtained by the investigator or a member of the study staff designated to conduct the consent process before any study procedures. An example participant consent form is provided as online supplemental file 3. Where participants lack capacity because of hepatic encephalopathy, sedation or mechanical ventilation, consent will be sought from a personal or professional consultee or legally authorised representative in accordance with the Mental Capacity Act 2005 (or equivalent legislation) and approved ethics procedures. Participants or their representatives will receive a clear explanation of the nature, scope and possible consequences of the study, in both oral and written form. For patients with limited English proficiency, where translated written study materials are unavailable, a professional interpreter will be used to facilitate the informed consent process.

### Study intervention

Participants randomised to the treatment arm will receive SoC plus up to seven daily DIALIVE 2.0 treatment sessions during a 10-day treatment window. DIALIVE 2.0 therapy is prescribed using a plasma filtration volume equivalent to 5% of the participant’s ideal body weight (IBW) with treatment sessions lasting 4 to 6 hours, allowing for planned or unplanned interruptions (eg, transfer for imaging, device clotting, vascular access issues). Dose was selected on the basis of preclinical data demonstrating effective clearance of albumin-bound toxins and inflammatory mediators. Blood flow rates are commenced at 50 mL/min and increased gradually over 30–60 min to 150–200 mL/min, depending on haemodynamic tolerance.

All participants will receive SoC consistent with the current European Association for the Study of the Liver (EASL) clinical practice guidelines on ACLF,^12^ and local critical care practice. This includes identification and treatment of precipitating events, appropriate antimicrobial therapy, vasopressors, renal replacement therapy, respiratory support, treatment of hepatic encephalopathy, management of bleeding, correction of coagulation abnormalities and nutritional support. Other ECLS devices will not be permitted during the study period. Renal replacement therapy may be initiated for conventional renal indications.

Replacement fluid is administered according to ultrafiltration losses, and clinical fluid balance targets. Albumin replacement equivalent to a total dose of 1–1.5 g/kg IBW of 20% HAS will be administered intravenously, with 40% of the total dose at the start of treatment and the remaining 60% infused during or at the end of the session. Mean arterial pressure will be continuously monitored and maintained above 65 mm Hg at all times. One pool of fresh frozen plasma will be given after each treatment session to mitigate coagulation-factor loss. Laboratory monitoring will be undertaken according to predefined study procedures, with senior clinical supervision throughout treatment.

Unfractionated heparin and/or epoprostenol, administered according to local protocols for renal replacement therapy, will be used to maintain circuit patency, with the choice and dose individualised according to the participant’s coagulation profile. Anticoagulation will be monitored with serial platelet counts and coagulation parameters, including international normalised ratio, activated partial thromboplastin time and fibrinogen. Regional citrate anticoagulation is not permitted. In the event of clinically significant bleeding, thrombocytopenia or excessive anticoagulation, DIALIVE 2.0 treatment may be temporarily interrupted and coagulation abnormalities corrected with platelets or clotting products as clinically indicated on a case-by-case basis. If heparin-induced thrombocytopenia is suspected, heparin will be discontinued and an alternative strategy to maintain circuit patency, including epoprostenol where appropriate, will be considered in accordance with local protocols.

Given the clinical heterogeneity and rapidly changing physiology of participants with ACLF, rigid mandatory numerical stopping thresholds for all clinical scenarios would be overly restrictive and may not adequately reflect bedside clinical judgement. The protocol identifies a number of clinical situations in which DIALIVE 2.0 treatment may require temporary interruption or modification, including haemodynamic instability or increasing vasopressor requirements, clinically significant bleeding or coagulopathy, thrombocytopenia, suspected haemolysis, hypersensitivity reactions, circuit or filter clotting, vascular-access complications and other clinically significant adverse events (AEs). Treatment may be temporarily interrupted where continuation is considered potentially unsafe and may be resumed once the precipitating issue has resolved or sufficiently improved, and the treating investigator considers further treatment to be safe. Permanent discontinuation will be considered where an event is severe, persistent or recurrent despite appropriate management, or where further DIALIVE 2.0 treatment is considered clinically contraindicated. All treatment interruptions, incomplete sessions, corrective measures, treatment delivered and reasons for permanent discontinuation will be documented.

### Evaluations during and after treatment

### Screening period

During screening, participants receive SoC while eligibility is determined. Written informed consent will be obtained. The data on which the eligibility decision is based, as well as other baseline data, will be collected.

### Randomisation (day 0)

After the screening visit and once local laboratory results are available, the investigator will reconfirm eligibility, randomise the participant and collect blood and urine samples. The first DIALIVE 2.0 treatment will be initiated within 24 hours of randomisation.

### Treatment period (days 1–10)

The treatment period extends from randomisation through 10 study days, or until ACLF resolution or a treatment-termination criterion (liver transplantation, a SAE requiring the cessation of DIALIVE therapy or death) is met. Each DIALIVE 2.0 session should last 4–6 hours. Treatment may be temporarily interrupted for clinical or technical reasons and resumed when considered safe. A cumulative treatment duration of at least 4 hours is required for a completed session—otherwise, the session will be recorded as incomplete, together with the treatment duration, reason for interruption and any corrective measures undertaken.

Participants who initially achieve the primary efficacy endpoint during DIALIVE 2.0 treatment but subsequently relapse (assessed ≥24 hours after the last DIALIVE 2.0 session) before the end of the 10-day window are eligible, in the absence of AEs or other exclusionary factors, for additional DIALIVE 2.0 sessions until ACLF resolution is re-established. The maximum number of DIALIVE 2.0 sessions per participant is capped at seven. Treatment cannot be initiated beyond day 9 (day 10 is the final treatment-period assessment). All participants will receive SoC throughout their hospital stay. For DIALIVE 2.0-treated participants, full blood count (FBC) and coagulation will be repeated 4 hours after treatment initiation and FBC, coagulation, liver function tests (LFTs) and urea and electrolytes (U&Es) at the end of each treatment.

### Post-treatment period

The post-treatment period begins at the end of the treatment period and continues until hospital discharge or day 28, whichever occurs first. During this period, study assessments will be performed weekly from day 14.

### Follow-up period

Participants will be followed up at 28 (±2) and 90 (±3) days. During this period, clinical status, therapeutic and diagnostic procedures and laboratory results will be collected and documented. Outcome data including dates of intensive care unit (ICU) and hospital discharge, transplantation status and final diagnoses will be recorded. For participants who are not hospitalised during follow-up and are unavailable for an outpatient review, telephone follow-up will be performed.

Participants who discontinue DIALIVE 2.0 treatment but remain enrolled in the study will continue follow-up through day 90 wherever possible, with scheduled outcome and safety data continuing to be collected. Participants withdrawn because of an AE or protocol non-compliance will continue to be followed for safety outcomes through day 90 where possible. No further protocol-mandated follow-up will be undertaken after withdrawal of consent.

The DIALIVE 2.0 study design is summarised in figure 2. Assessment procedures will be conducted as outlined in table 2. At day 90, AEs and outcomes will be reviewed.

**Figure 2:**
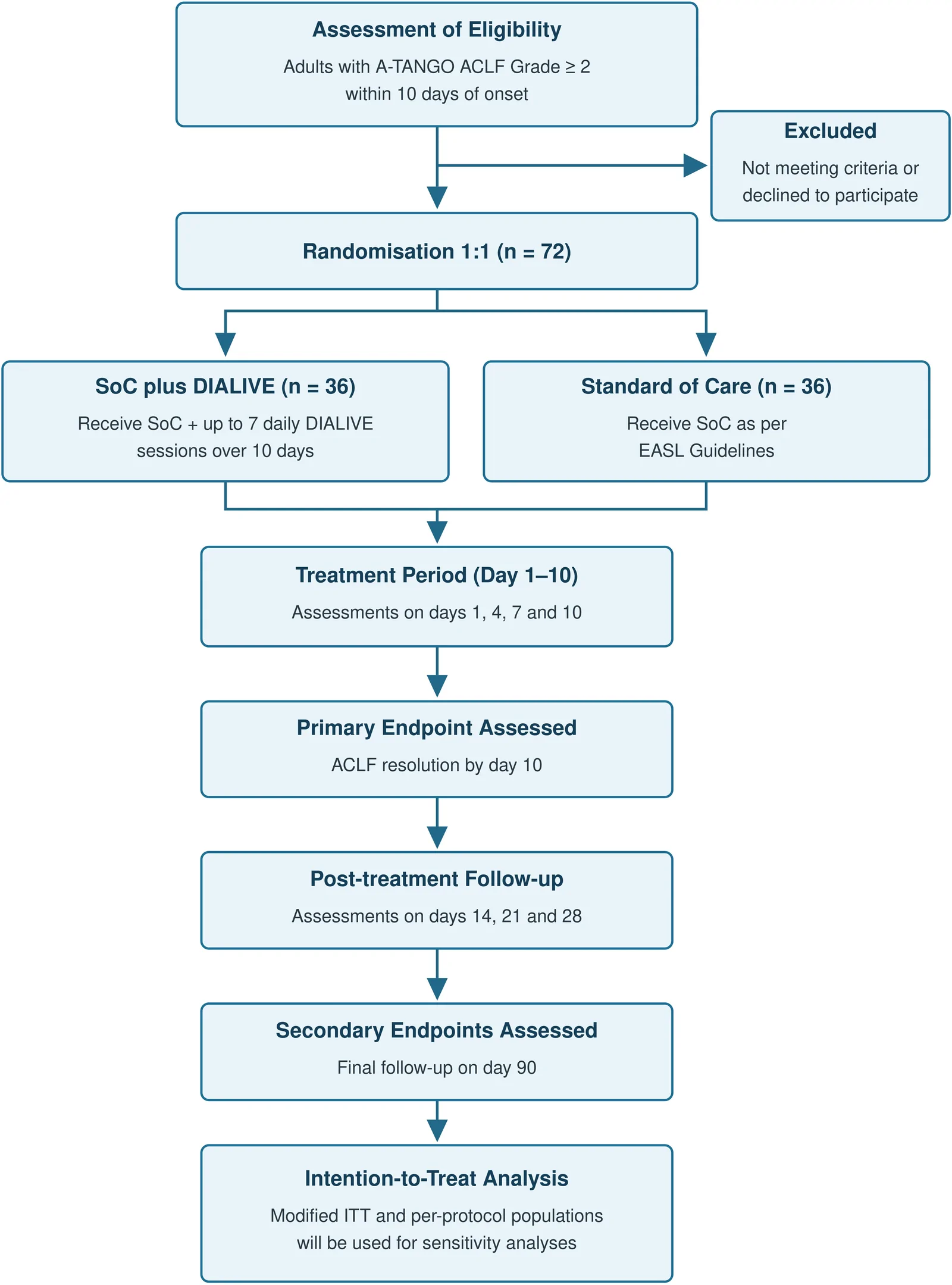
DIALIVE 2.0 study design. ACLF, acute-on-chronic liver failure; EASL, European Association for the Study of the Liver; ITT, intention-to-treat; SoC, standard of care.

**Table 2:** Assessment procedures

Schedule	Assessment
Days 1, 4, 7, 10 and 28	Concomitant medication review
	Physical examination
	Weight and body mass index
	Vital signs
	Ascites assessment
	Assessment of hepatic encephalopathy (West-Haven criteria)
	12-lead ECG
	Routine haematology, coagulation and biochemistry
	Infection surveillance
	A-TANGO OF score, CLIF-C OF score, MELD score and Child-Pugh score
	A-TANGO ACLF-WBC score (on day 28 only)
	Adverse-event review
Days 14 and 21	Vital signs
	Assessment of hepatic encephalopathy
	Routine haematology, coagulation and biochemistry
	A-TANGO OF score, CLIF-C OF score, MELD score and Child-Pugh score

ACLF, acute-on-chronic liver failure; CLIF-C, chronic liver failure-consortium; MELD, model for end-stage liver disease; OF, organ failure; WBC, white blood cell count.

### Monitoring

An independent Data Safety Monitoring Board (DSMB), comprising a hepatologist, intensivist and statistician, will oversee participant safety and trial conduct. Interim safety reviews will be undertaken after enrolment of participants 5, 15, 30, 45 and 60. The DSMB will review safety and efficacy signals, advise on protocol modifications where required, and may recommend study suspension or termination if unacceptable safety concerns or compelling treatment effects are identified.

All AEs, SAEs and unanticipated adverse device effects (UADEs) will be recorded and reported in accordance with Medicines and Healthcare products Regulatory Agency (MHRA) requirements. An initial MHRA safety and performance review will be undertaken after the first three participants have completed DIALIVE 2.0 treatment. Screening may continue during the review period, but no further participants will be randomised until the review is complete. Ongoing safety oversight will include expedited reporting of SAEs and UADEs, periodic safety updates and reporting of protocol deviations in line with applicable regulatory requirements.

## Analysis

### Primary endpoint

The primary efficacy endpoint is the proportion of participants achieving ACLF resolution by day 10 in the DIALIVE 2.0 plus SoC group, compared with the SoC group. ACLF resolution is defined as the participant meeting the A-TANGO criteria for no ACLF at the day 10 assessment.

### Secondary endpoints

Secondary efficacy endpoints:

1. Transplant-free survival at 28 days post-enrolment (defined as time to death or liver transplantation, whichever occurs first), compared between treatment arms.
2. Transplant-free survival at 90 days post-enrolment (defined as time to death or liver transplantation, whichever occurs first), compared between treatment arms.
3. Predicted 6-month and 1-year survival from baseline versus day 28, as judged by the A-TANGO ACLF-WBC score.
4. Time to ICU and hospital discharge, with death treated as a competing risk and censoring at transplantation or transfer to another facility.
5. Proportion of participants with resolution of individual organ failures at day 28 versus baseline, assessed using the A-TANGO ACLF subscores (death, liver transplantation or other drop out will be counted as non-improvement).

Safety endpoints:

The trial is also designed to demonstrate that DIALIVE 2.0 plus SoC does not increase the incidence of SAEs over the first 4 weeks and 3 months post-enrolment. Specific safety endpoints include:

1. Incidence of SAEs.
2. Incidence of device-related SAEs.
3. Device deficiencies.
4. Time to onset and resolution of device-related SAEs.

Supportive prognostic analysis:

Transplant-free survival at 28 days in participants with resolution of ACLF within 10 days, versus participants with persistent ACLF at day 10 irrespective of treatment.

### Additional assessments

Health-economic data including number of hospital admissions, AE counts, length of ICU stay, mortality outcomes, extrapolated quality-of-life data and total costs by intervention and comparator will be collected to develop a health-economic model of SoC plus DIALIVE 2.0 versus SoC alone.

Biomarkers listed in table 3 will be measured in samples collected on days 0, 4, 10 and 28 to assess systemic inflammation, albumin function, cell death and renal injury.

**Table 3:** Biomarker collection

Specimen	Biomarker assessments
Blood	Systemic inflammation: IL-1β, IL-6, IL-8, IL-10, TNF-α, CCL5, soluble CD163; together with transcriptomic and metabolomic profiling
	Albumin antioxidant function: HMA, HNA1, HNA2, ischaemia-modified albumin, detoxification capacity
	Cell-death markers: M30, M65, RIPK3, HMGB1, GSDMD
	TLR4/IL-1β/IL-18 reporter cell assay
Urine	Renal injury: neutrophil gelatinase-associated lipocalin

### Data collection and storage

The sponsor will provide materials together with instructions for collection, processing, storage and shipment of samples. Samples will be stored at the investigational site under restricted access until shipment to the core laboratory at the end of the study, will remain pseudonymised throughout and will be destroyed after analysis. All data will be collected and stored in pseudonymised form using a secure eCRF and a centralised database, in compliance with applicable data-protection legislation.

### Statistical analysis

All randomised participants will be included in the intention-to-treat (ITT) population. Unless otherwise specified, primary and secondary efficacy endpoints will be analysed in the ITT population. The safety population will include all participants who commence the treatment strategy to which they were randomised. Modified ITT (mITT) and per-protocol (PP) populations will be used for sensitivity analyses. The mITT population will comprise all randomised participants who receive at least 1 day of their allocated treatment strategy. Participants who die before day 10 without a post-baseline assessment will remain in the mITT population and will be classified as non-responders. The PP population will comprise participants who met all eligibility criteria, received at least 1 day of the treatment strategy to which they were randomised, and underwent at least two post-randomisation evaluations. Analyses will be performed using R V.4.4.0 or later^13^ (R Core Team, 2025). Continuous variables will be analysed using parametric or non-parametric methods as appropriate, and categorical variables using Fisher’s exact or χ^2^ tests. A two-sided p<0.05 will be considered statistically significant.

The primary endpoint, ACLF resolution by day 10, will be analysed using logistic regression. Death before day 10 and liver transplantation before day 10 without prior ACLF resolution will count as non-resolution. The absolute risk difference and 95% CI will be the primary effect measure, with OR and relative risk also reported. Prespecified sensitivity analyses will (1) exclude participants undergoing liver transplantation before day 10 and (2) repeat the primary analysis in the mITT and PP populations.

If the primary endpoint is statistically significant, 28-day transplant-free survival will be tested within a fixed-sequence procedure controlling the family-wise error rate at a two-sided 0.05 level. The absolute difference in 28-day transplant-free survival and 95% CI will be reported, together with an HR from a Cox model stratified by baseline A-TANGO ACLF grade. The trial sample size is determined by the primary endpoint, with no separate power calculation performed for 28-day transplant-free survival. All remaining secondary efficacy endpoints will be considered supportive and reported with 95% CIs without formal hypothesis testing or multiplicity adjustment. Time to ICU and hospital discharge will be analysed using cumulative incidence methods, with death as a competing risk. Organ failure resolution will be compared between groups as proportions, with death and liver transplantation classified as non-improvement. Changes in predicted 6-month and 1-year survival from baseline to day 28 will be compared between treatment arms, adjusting for baseline values.

A prespecified landmark analysis will assess whether ACLF resolution by day 10 is prognostic for subsequent transplant-free survival. Among participants alive and evaluable at day 10, transplant-free survival from day 10 to day 28 will be compared between those with ACLF resolution and those with persistent ACLF, irrespective of treatment allocation. As classification is based on a postrandomisation outcome, this analysis does not estimate the randomised treatment effect and will be reported with an effect estimate and 95% CI as a supportive, non-confirmatory analysis.

### Missing data

Missing data will be handled according to prespecified endpoint-specific rules. For the primary endpoint, death and liver transplantation before day 10 will be handled as intercurrent events under the composite strategy and not considered missing data. No imputation will be performed for participants who withdraw consent; the extent and reasons for missing data will be summarised by treatment arm. For laboratory measures, the last value recorded within 24 hours before death will be retained as the final observed value. No values will be carried forward after death, withdrawal of consent or liver transplantation.

### Ethics and dissemination

The study will be conducted in accordance with the Declaration of Helsinki, Good Clinical Practice and applicable UK regulatory requirements. Regulatory approval has been obtained from the MHRA, which issued a 'no objection’ decision (CI/2025/0075/GB). A favourable opinion was obtained from the London–South East Research Ethics Committee (26/LO/0090), and Health Research Authority approval was granted before study initiation. The study will be conducted in compliance with the Medical Devices Regulations 2002.

The trial is sponsored by Yaqrit, which is responsible for study oversight, regulatory compliance and provision of the investigational device. The study was designed collaboratively by the sponsor and academic investigators. Results will be disseminated through peer-reviewed publications, conference presentations and stakeholder engagement.

Important protocol amendments will be prepared by the sponsor and submitted to the relevant ethics and regulatory authorities as required. Approved amendments will be communicated to participating investigators. Where new information may affect participant safety, participants will be informed and re-consented where necessary. No trial-specific ancillary or post-trial care is planned beyond ongoing standard clinical care. Insurance coverage is in place to provide compensation in the event of harm resulting from trial participation.

### Patient and public involvement

Patient and public involvement was embedded throughout the study design, starting at the funding application stage. Patients with lived experience of ACLF contributed to the following elements : development of research questions, selection of outcomes, optimisation of recruitment strategy and participant materials. They will continue to support recruitment and dissemination activities.

## Discussion

This trial asks whether DIALIVE 2.0 can move a meaningful proportion of patients with established ACLF from a trajectory of multiorgan failure to one of clinical recovery within 10 days. The question matters because, after three decades of work on ECLS, no device has yet improved survival in a randomised trial. MARS, Prometheus and ELAD each generated genuine enthusiasm in early-phase work, and each failed at the randomised trial endpoint.^5–7^ All three trials used 28-day or 90-day all-cause mortality. Mortality matters most to patients, but it is a difficult primary endpoint in ACLF: liver transplantation is a competing risk, drop-out is high and the absolute event rate varies sharply between cohorts and over time. The trials were therefore either underpowered for plausible effect sizes or powered on optimistic assumptions, and none used an endpoint that captured the disease-modifying biology of the intervention. The observation that early ACLF resolution is independently associated with improved 28-day and 90-day survival^3^ gives a far more practical proof-of-efficacy endpoint.

The Chronic Liver Failure Consortium (CLIF-C) organ failure score and the derived CLIF-C ACLF prognostic score^14^ have transformed how ACLF is defined and stratified in clinical practice. For this trial, however, the original CLIF scoring system has limitations. It was derived in a largely European population, whose dominant precipitants were alcohol and bacterial infection and may discriminate less well in settings where viral hepatitis or metabolic dysfunction-associated steatohepatitis are the leading precipitants.^4 15^ It groups all patients with three or more organ failures into a single category and so does not separate the highest-risk group of patients with four or more failing organs, in whom any rescue therapy has very limited biological room to act. The cut-offs for some individual components are also not well aligned with the points at which ECLS is clinically deployable or likely to be useful. The A-TANGO Organ Failure score^11^ was developed and validated across geographically diverse cohorts that include the full range of contemporary ACLF precipitants, and was explicitly designed with clinical trials in mind. It refines the organ-failure thresholds, recognises ACLF grade 4 as a distinct category and is paired with the A-TANGO ACLF-WBC and A-TANGO ACLF-CRP prognostic scores, which incorporate white blood cell count and C-reactive protein, respectively (online supplemental file 2). Because A-TANGO provides a framework for assessing ACLF resolution, it enables this trial to use a clinically meaningful, mechanism-aligned primary endpoint rather than all-cause mortality.

Day-10 ACLF resolution is the answer to a practical question: what is the smallest and earliest signal that would tell us whether DIALIVE 2.0 is changing the course of disease? It is the period over which the on-treatment biology of the device must translate into reversal of organ dysfunction, or not. It is the period over which resolvers and non-resolvers diverge in their 28-day and 90-day survival.^3^ Moreover, it is achievable within the sample size that can realistically be recruited in the UK over the funded duration of the trial. The supportive prognostic analysis of 28-day transplant-free survival according to ACLF resolution status at day 10 is included to assess the clinical relevance of early ACLF resolution. It provides evidence for the prognostic relevance of ACLF resolution as a clinically meaningful treatment-response endpoint and links early improvement in organ failure with a hard patient-centred outcome. The endpoint strategy prioritises patient relevance by placing 28-day transplant-free survival as the key secondary endpoint within the confirmatory testing sequence. A trial that is positive on the primary endpoint and shows consistent signals on the secondary endpoints would make a clear case for a confirmatory, mortality-endpoint pivotal trial. A clearly negative trial, paired with the embedded biomarker programme, would tell us whether the SPEAR concept is worth developing further and, if so, in which patient subgroups.

Patient selection is the central clinical judgement in this field. We have set the inclusion thresholds to capture patients who are sick enough to need an extracorporeal intervention but who retain enough biological reserve to respond, and we have excluded patients with extreme physiological derangement for safety reasons. We accept that this leaves some clinically deserving patients outside the trial—patients on high-dose vasopressors, those with established invasive fungal infection or severe disseminated intravascular coagulation and the very elderly—and that any pragmatic real-world adoption of DIALIVE 2.0 will need further studies in these groups.

## Supplementary material

10.1136/bmjopen-2026-125155 online supplemental file 1

10.1136/bmjopen-2026-125155 online supplemental file 2

10.1136/bmjopen-2026-125155 online supplemental file 3
