# Supplementary material for: Multicentre randomised controlled trial to evaluate the efficacy of DIALIVE 2.0, a liver dialysis device, for the treatment of acute-on-chronic liver failure: study protocol
Source: BMJ Open. 2026 Nov 6;16(11):e125155. doi: 10.1136/bmjopen-2026-125155 (PMC13756337; doi:10.1136/bmjopen-2026-125155)
Supplement: Supplementary file 1 [file bmjopen-2026-125155.reviewer_comments.pdf]

PEER REVIEW HISTORY

BMJ Open publishes all reviews undertaken for accepted manuscripts. Reviewers are asked to complete a checklist review form and are provided with free text boxes to elaborate on their assessment. These free text comments are reproduced below.

ARTICLE DETAILS

Title (Provisional)

Study protocol for a multicentre randomised controlled trial to evaluate the efficacy of DIALIVE 2.0, a liver dialysis device, for the treatment of acute-on-chronic liver failure

Authors

Jameie-Oskooei, Sina; Saha, Rohit; Patel, Sameer; Bangash, Mansoor N; Mehta, Gautam; Morgan, Carrie; Carbonell, Juan-Antonio; Agarwal, Banwari; Jalan, Rajiv

---

VERSION 1 - REVIEW

**Reviewer**	**1**
**Name**	**Zhang, Ling**
**Affiliation**	**Sichuan University West China Hospital, Nephrology**
**Date**	**26-Jul-2026**
**COI**	**none**

This manuscript describes the protocol for a multicenter, randomised controlled trial evaluating DIALIVE 2.0 in patients with acute-on-chronic liver failure (ACLF). This is a clinically important study addressing a major unmet need, as effective disease-modifying therapies for ACLF remain limited and the condition is associated with substantial short-term mortality. The manuscript is generally well structured and provides a clear rationale for the trial. However, several important aspects of the comparator, outcome definitions, participant selection and treatment safety procedures require further clarification to ensure consistency across participating centers. I recommend that the authors address the following major comments before publication.

1. Definition and standardization of standard of care. The control group is described as receiving standard of care (SoC) according to the European Association for the Study of the Liver guidelines. However, the specific components of SoC are not sufficiently described. It is important to clarify whether other extracorporeal liver-support therapies are prohibited, permitted as concomitant treatment, or allowed only as rescue therapy. If such treatments are permitted, the corresponding indications and their handling in the efficacy analysis should be prespecified.

2. Operational definition of ACLF resolution. The primary endpoint is the proportion of participants achieving ACLF resolution by day 10. However, the definition of “ACLF resolution” is not clearly stated. The manuscript does not explicitly state whether resolution requires transition to no A-TANGO ACLF, improvement to A-TANGO ACLF grade 1, or another prespecified degree of improvement.

3. Definition of non-response during the pre-randomisation period. Eligibility requires ACLF to be non-responsive to SoC for up to 48 hours before randomisation. The manuscript qualitatively describes this population as patients whose ACLF “progresses, plateaus or relapses” despite SoC, but no quantitative criteria are provided. For example, it should be clarified whether non-response is defined by a lack of reduction in the A-TANGO organ-failure score or ACLF grade, or failure of bilirubin.

4. Definition and analysis of 28-day transplant-free survival. One secondary endpoint is described as “28-day transplant-free survival in participants with ACLF resolution by day 10 versus participants with persistent ACLF at day 10, in each arm.” This analysis classifies participants according to a post-randomisation outcome. It therefore primarily evaluates the prognostic association between day-10 ACLF resolution and subsequent survival. It cannot, by itself, provide an unbiased estimate of the randomised treatment effect of DIALIVE 2.0 versus SoC. I suggest that the principal efficacy analysis of 28-day transplant-free survival should compare the two randomised treatment groups directly.

5. Criteria for temporary interruption and permanent discontinuation of DIALIVE 2.0. The manuscript states that mean arterial pressure will be maintained above 65 mmHg and that treatment may be interrupted because of clinical reasons, vascular-access problems or filter clotting. It also refers to treatment-termination criteria and stopping rules, but the specific participant-level criteria are not provided.

The protocol should distinguish between criteria for temporarily interrupting a treatment session and criteria for permanently discontinuing DIALIVE 2.0. Prespecified criteria should address clinically relevant situations such as sustained hypotension or increasing vasopressor requirements, major bleeding, clinically significant deterioration in coagulation parameters, severe thrombocytopenia, filter or circuit clotting, vascular-access complications, suspected haemolysis, severe allergic reactions and other serious device-related adverse events. Criteria for resuming treatment after an interruption and the handling of incomplete treatment sessions should also be described.

6. The anticoagulation strategy for the extracorporeal circuit is not reported. This is an important safety and feasibility consideration because patients with ACLF frequently have complex haemostatic abnormalities and may be at risk of both bleeding and circuit thrombosis. The authors should describe the anticoagulation regimen, including the monitoring method. The alternative strategy for participants who develop bleeding, thrombocytopenia or suspected heparin-induced thrombocytopenia should also be specified.

VERSION 1 - AUTHOR RESPONSE

Reviewer's Comments

Comment 1: Definition and standardization of standard of care. The control group is described as receiving standard of care (SoC) according to the European Association for the Study of the Liver guidelines. However, the specific components of SoC are not sufficiently described. It is important to clarify whether other extracorporeal liver-support therapies are prohibited, permitted as concomitant treatment, or allowed only as rescue therapy. If such treatments are permitted, the corresponding indications and their handling in the efficacy analysis should be prespecified.

Response: We have revised the manuscript to clarify the principal components of standard of care (SoC), which include identification and treatment of precipitating events, appropriate antimicrobial therapy, vasopressor support, renal replacement therapy when clinically indicated, respiratory support, treatment of hepatic encephalopathy, management of bleeding and coagulation abnormalities, and nutritional support. Although a fully prescriptive description of SoC is beyond the scope of the manuscript, these measures will be delivered in accordance with current EASL guidance (now cited in the manuscript) and local clinical practice. Importantly, concomitant use of other extracorporeal liver-support therapies will not be permitted during the study period. Renal replacement therapy may be initiated or continued for conventional renal indications and is considered part of SoC rather than an alternative liver-support intervention.

Comment 2: Operational definition of ACLF resolution. The primary endpoint is the proportion of participants achieving ACLF resolution by day 10. However, the definition of "ACLF resolution" is not clearly stated. The manuscript does not explicitly state whether resolution requires transition to no A-TANGO ACLF, improvement to A-TANGO ACLF grade 1, or another prespecified degree of improvement.

Response: We thank the reviewer for highlighting the need for a precise operational definition. We have revised the manuscript to clarify that ACLF resolution is defined as transition from A-TANGO ACLF grade 2–4 at baseline to no ACLF according to the A-TANGO criteria by the Day-10 assessment. Improvement to A-TANGO ACLF grade 1 alone is therefore not considered ACLF resolution for the primary endpoint.

Comment 3: Definition of non-response during the pre-randomisation period. Eligibility requires ACLF to be non-responsive to SoC for up to 48 hours before randomisation. The manuscript qualitatively describes this population as patients whose ACLF "progresses, plateaus or relapses" despite SoC, but no quantitative criteria are provided. For example, it should be clarified whether non-response is defined by a lack of reduction in the A-TANGO organ-failure score or ACLF grade, or failure of bilirubin.

Response: The protocol has been revised to require participants to have received at least (rather than up to) 48 hours of appropriate standard of care before eligibility is reassessed. Non-response will be determined by repeat assessment using the A-

TANGO criteria, and participants will be considered non-responsive if they continue to meet criteria for A-TANGO ACLF grade 2–4 after this period. Non-response is therefore defined by persistence of A-TANGO ACLF grade 2–4, rather than by failure of an individual laboratory parameter, such as bilirubin, to improve.

Comment 4: Definition and analysis of 28-day transplant-free survival. One secondary endpoint is described as "28-day transplant-free survival in participants with ACLF resolution by day 10 versus participants with persistent ACLF at day 10, in each arm." This analysis classifies participants according to a post-randomisation outcome. It therefore primarily evaluates the prognostic association between day-10 ACLF resolution and subsequent survival. It cannot, by itself, provide an unbiased estimate of the randomised treatment effect of DIALIVE 2.0 versus SoC. I suggest that the principal efficacy analysis of 28-day transplant-free survival should compare the two randomised treatment groups directly.

Response: We thank the reviewer for this important comment and agree that the principal analysis of 28-day transplant-free survival should compare the two randomised treatment groups directly. We have therefore made 28-day transplant-free survival between the DIALIVE 2.0 plus SoC and SoC groups as the main secondary efficacy endpoint. We have retained the analysis of 28-day transplant-free survival according to Day-10 ACLF resolution status as a supportive prognostic analysis. Its purpose is to assess the clinical relevance of early ACLF resolution by determining whether participants who achieve resolution of ACLF within 10 days have better subsequent transplant-free survival than those with persistent ACLF at Day 10. This analysis is not intended to estimate the randomised treatment effect, but rather to establish whether early ACLF resolution is associated with a clinically meaningful, patient-centred outcome. The statistical analysis plan has been amended accordingly to reflect these changes.

Comment 5: Criteria for temporary interruption and permanent discontinuation of DIALIVE 2.0. The manuscript states that mean arterial pressure will be maintained above 65 mmHg and that treatment may be interrupted because of clinical reasons, vascular-access problems or filter clotting. It also refers to treatment-termination criteria and stopping rules, but the specific participant-level criteria are not provided. The protocol should distinguish between criteria for temporarily interrupting a treatment session and criteria for permanently discontinuing DIALIVE 2.0. Prespecified criteria should address clinically relevant situations such as sustained hypotension or increasing vasopressor requirements, major bleeding, clinically significant deterioration in coagulation parameters, severe thrombocytopenia, filter or circuit clotting, vascular-access complications, suspected haemolysis, severe allergic reactions and other serious device-related adverse events. Criteria for resuming treatment after an interruption and the handling of incomplete treatment sessions should also be described.

Response: We agree that the distinction between temporary interruption and permanent discontinuation requires clarification. Given the clinical heterogeneity and rapidly changing physiology of patients with ACLF, we considered that rigid mandatory numerical stopping thresholds for all clinical scenarios could be unnecessarily restrictive and may not appropriately reflect bedside clinical judgement. We have therefore revised the protocol to identify a number of clinically

important situations that should prompt consideration of temporary interruption. These include haemodynamic instability, bleeding/coagulopathy, thrombocytopenia, circuit clotting, vascular-access complications, suspected haemolysis, hypersensitivity reactions and other significant adverse events. Decisions will remain the responsibility of the treating investigator, taking into account the severity, persistence and reversibility of the event and the participant's overall clinical condition. We have also clarified the recording and handling of interrupted and incomplete treatment sessions. Each DIALIVE 2.0 session should last 4–6 hours. Treatment may be temporarily interrupted for clinical or technical reasons and resumed when considered safe. A cumulative treatment duration of at least 4 hours is required for a completed session; otherwise, the session will be recorded as incomplete, together with the treatment duration, reason for interruption and any corrective measures undertaken.

Comment 6: The anticoagulation strategy for the extracorporeal circuit is not reported. This is an important safety and feasibility consideration because patients with ACLF frequently have complex haemostatic abnormalities and may be at risk of both bleeding and circuit thrombosis. The authors should describe the anticoagulation regimen, including the monitoring method. The alternative strategy for participants who develop bleeding, thrombocytopenia or suspected heparin-induced thrombocytopenia should also be specified.

Response: We thank the reviewer for highlighting this important safety consideration. We have revised the manuscript to clarify the anticoagulation strategy for the DIALIVE 2.0 extracorporeal circuit. Unfractionated heparin and/or epoprostenol will be administered to maintain circuit patency, with the choice and dose determined by the participant's coagulation profile and according to established local protocols for renal replacement therapy. Anticoagulation will be monitored according to standardised local heparin protocols, together with serial assessment of platelet count and coagulation parameters, including INR, APTT and fibrinogen. Regional citrate anticoagulation is not permitted. If clinically significant bleeding, excessive anticoagulation or significant thrombocytopenia develops, anticoagulation and/or DIALIVE 2.0 treatment may be temporarily interrupted and the underlying abnormality managed, including administration of platelets or coagulation products where clinically indicated; treatment may subsequently be resumed when the abnormality has sufficiently resolved, and the treating investigator considers this safe. Participants with known heparin-induced thrombocytopenia (HIT) type II are excluded at enrolment. If HIT is suspected, heparin will be discontinued and an alternative strategy to maintain circuit patency, including epoprostenol where appropriate, will be considered in accordance with local protocols.

VERSION 2 - REVIEW

**Reviewer**	**1**
**Name**	**Zhang, Ling**
**Affiliation**	**Sichuan University West China Hospital, Nephrology**
**Date**	**08-Oct-2026**

COI

None.
