# Supplementary material for: Multicentre randomised controlled trial to evaluate the efficacy of DIALIVE 2.0, a liver dialysis device, for the treatment of acute-on-chronic liver failure: study protocol
Source: BMJ Open. 2026 Nov 6;16(11):e125155. doi: 10.1136/bmjopen-2026-125155 (PMC13756337; doi:10.1136/bmjopen-2026-125155)
Supplement: Supplementary file 2 [file bmjopen-16-11-s001.docx]

**A-TANGO Organ Failure (A-TANGO OF) Grading System**

Adapted from Engelmann et al. [11], licensed under Creative Commons Attribution (CC BY 4.0)

| **Organ System** | **Subscore 1** | **Subscore 2** | **Subscore 3 (Organ Failure)** |
| --- | --- | --- | --- |
| Liver | Bilirubin < 6 mg/dL | Bilirubin ≥ 6 and < 20 mg/dL | Bilirubin ≥ 20 mg/dL |
| Kidney | Creatinine < 1.4 mg/dL | Creatinine ≥ 1.4 and < 2 mg/dL, or terlipressin | Creatinine ≥ 2 mg/dL, RRT, or AKI 1-B/2/3 |
| Brain | Grade 0–1 (West-Haven) | Grade 2 | Grade 3–4, or mechanical ventilation for cerebral failure |
| Coagulation | INR < 1.6 | INR ≥ 1.6 and < 2.2 | INR ≥ 2.2 |
| Circulation | MAP ≥ 70 mmHg | MAP < 70 mmHg | Vasopressor use for circulatory failure |
| Respiration | PaO₂/FiO₂ > 350 or SpO₂/FiO₂ > 440 | PaO₂/FiO₂ > 225 and ≤ 350, or SpO₂/FiO₂ > 325 and ≤ 440 | PaO₂/FiO₂ ≤ 225, SpO₂/FiO₂ ≤ 325, or mechanical ventilation for respiratory failure |

Individual organ failures are defined by an A-TANGO sub-score of 3. ACLF grades are defined as follows:

- No A-TANGO ACLF: patients without liver, kidney, coagulation or respiratory failure, or with isolated brain or circulatory failure without reaching sub-score 2 in any other organ.
- A-TANGO ACLF grade 1: a single liver, kidney, coagulation or respiratory failure.
- A-TANGO ACLF grade 2: two organ failures (sub-score 3).
- A-TANGO ACLF grade 3: three organ failures (sub-score 3).
- A-TANGO ACLF grade 4: four or more organ failures.

Abbreviations: AKI, acute kidney injury; INR, international normalised ratio; MAP, mean arterial pressure; PaO₂/FiO₂, ratio of arterial oxygen partial pressure to fractional inspired oxygen; RRT, renal replacement therapy; SpO₂/FiO₂, ratio of pulse-oximetric oxygen saturation to fractional inspired oxygen. AKI stages are defined according to the revised International Club of Ascites (ICA) consensus criteria for acute kidney injury in cirrhosis.
