# Supplementary material for: Multicentre randomised controlled trial to evaluate the efficacy of DIALIVE 2.0, a liver dialysis device, for the treatment of acute-on-chronic liver failure: study protocol
Source: BMJ Open. 2026 Nov 6;16(11):e125155. doi: 10.1136/bmjopen-2026-125155 (PMC13756337; doi:10.1136/bmjopen-2026-125155)
Supplement: Supplementary file 3 [file bmjopen-16-11-s002.docx]

**A-TANGO Clinical Score Formulas**

Reproduced from Engelmann et al. [11], licensed under Creative Commons Attribution (CC BY 4.0)

A-TANGO ACLF-CRP = 10 × (0.30219 × A-TANGO OF score + 0.03351 × age + 0.29446 × log(CRP)) − 9

A-TANGO ACLF-WBC = 10 × (0.30205 × A-TANGO OF score + 0.03313 × age + 0.45920 × log(WBC)) − 8
