# Supplementary material for: Multicentre randomised controlled trial to evaluate the efficacy of DIALIVE 2.0, a liver dialysis device, for the treatment of acute-on-chronic liver failure: study protocol
Source: BMJ Open. 2026 Nov 6;16(11):e125155. doi: 10.1136/bmjopen-2026-125155 (PMC13756337; doi:10.1136/bmjopen-2026-125155)
Supplement: Supplementary file 4 [file bmjopen-16-11-s003.docx]

| **Study Title:** A Multi-Centre, Randomised Controlled Trial to Evaluate the Efficacy of DIALIVE 2.0, a Liver Dialysis Device, for the Treatment of Acute-on-Chronic Liver Failure (ACLF) Compared to Standard of Care | |
| --- | --- |
| **Protocol Number:** YAQ002-ES-1 | **Consent Form Version:** 5.0, 26^th^ February 2026 |
| **Investigator Name:** | |
| **Investigator Site:** | |
| **Centre Number:** | |
| **Patient Identification Number for this study:** | |
| **Sponsor:** Yaqrit Ltd. | |
| **IRAS ID:** 353362 | |

CONSENT FORM

|  | ***Please tick the boxes below*** |
| --- | --- |
| 1) I confirm that I have read the information sheet version _._, dated __/__/____ for the above study. I have had the opportunity to consider the information, ask questions and have had these answered satisfactorily. | ☐Yes ☐ No |
| 2) I understand that my participation is voluntary and that I am free to withdraw at any time without giving any reason, without my medical care or legal rights being affected. | ☐Yes ☐ No |
| 3) I understand that specific blood and urine samples will be transferred to a central Biobank in the UK for analysis. I agree to such transfer of samples. | ☐Yes ☐ No |
| 4) I accept that, if needed, additional blood samples for the analysis can be repeated. | ☐Yes ☐ No |
| 5) I understand that relevant sections of my medical notes and data collected during the study may be looked at by individuals from the sponsor company Yaqrit Ltd (or their representatives), from regulatory authorities or from the NHS Trust, where it is relevant to my taking part in this research. I give permission for these individuals to have access to my records. | ☐Yes ☐ No |
| 6) I understand that the information collected about me will be used to support other research in the future and may be shared anonymously with other researchers | ☐Yes ☐ No |
| 7) I agree to my General Practitioner being informed of my participation in the study. | ☐Yes ☐ No |
| 8) I understand that relevant sections of my medical records and data collected during the study may be reviewed by authorised members of the research team, the study sponsor, and regulatory authorities where relevant to my participation in this research. | ☐Yes ☐ No |
| 9) I understand and agree that my anonymised clinical data could be transferred to other countries in compliance with GDPR. | ☐Yes ☐ No |

| 10) I agree to take part in the above study. | ☐Yes ☐ No |
| --- | --- |
| 11) I confirm that I have received a copy of the entire Patient Information Sheet, and this signed consent form. | ☐Yes ☐ No |

Name of Participant	Signature	Date

I hereby declare that I have informed the above study participant verbally and in writing about the nature, significance, implications and risks of the study named above and that the individual has not been coerced into giving consent. A copy of the Information Sheet and this signed Consent Form has been provided to the participant, and one original has been filed in the site file.

Name of person taking consent	Signature	Date

*(Listed on delegation log)*

Investigator’s Name	Signature	Date
